# Efficacy and safety of therapeutic plasma exchange in stiff person syndrome

**DOI:** 10.1515/med-2021-0220

**Published:** 2021-03-30

**Authors:** Piotr F. Czempik, Justyna Gawryluk, Agnieszka Wiórek, Ewa Krzystanek, Łukasz J. Krzych

**Affiliations:** Department of Anaesthesiology and Intensive Care, Faculty of Medical Sciences in Katowice, Medical University of Silesia, Medyków 14, Katowice, 40-752, Poland; Department of Neurology, Faculty of Medical Sciences in Katowice, Medical University of Silesia, Medyków 14, Katowice, 40-752, Poland

**Keywords:** glutamic acid decarboxylase, procedure efficacy, procedure safety, stiff person syndrome, therapeutic plasma exchange

## Abstract

The stiff person syndrome (SPS) is an extremely rare neurological disorder with primarily immune-mediated etiology. The cardinal symptoms are progressive, fluctuating axial/proximal limb muscle stiffness and spasms. The diagnosis is based on the clinical picture, electromyography examination and detection of antibodies to glutamic acid decarboxylase (anti-GAD). Adverse effects of medications might preclude its use or increase in dosing, therefore symptomatic and/or immunomodulatory medical therapy might be ineffective in acute exacerbation of the disease. We present a case of a 49-year-old female with exacerbation of SPS, in whom some standard pharmacotherapy could not be introduced (clonazepam, baclofen used in the past) and doses of existing standard medications could not be increased (diazepam, gabapentin, and levetiracetam) due to adverse effects. Moreover, a newly introduced medication (methylprednisolone) also led to a serious adverse effect (severe hyperglycemia). The patient underwent therapeutic plasma exchange (TPE) with good effect and no complications. We review the literature regarding the efficacy and safety profile of TPE in exacerbation of SPS unresponsive to medical therapy. The procedure seems to have a good safety profile as an adjunct therapy for exacerbation of SPS not responding to standard medical therapy in this patient population.

## 1 Introduction

The stiff person syndrome (SPS) is a rare neurological disorder first reported in 1956 [1]. The incidence of the disease is 1/1,000,000, and it affects mostly women of 40–50 years of age [2]. SPS seems to have an autoimmune etiology, although its exact pathophysiologic mechanism remains unclear [3]. Most patients develop antibodies to the 65-kD isoform of glutamic acid decarboxylase (anti-GAD65), leading to a decrease in gamma-aminobutyric acid (GABA) [4]. Antibodies have been found to numerous other proteins: amphiphysin [5], gephyrin [6], Ri [7], dipeptidyl-peptidase-like protein-6, GABA_A_ receptor-associated protein (GABA_A_RAP), glycine receptor, and glycine transporter 2 [8]. The classic form of anti-GAD65-positive SPS is associated with other autoimmune disorders: insulin-dependent diabetes mellitus, Hashimoto’s thyroiditis, megaloblastic anemia, and celiac disease [9]. In the paraneoplastic form of SPS, associated with different types of cancers (breast, lung, thymus, colon, and lymphoma), antibodies to amphiphysin have been detected [5]. The typical presentation is progressive and fluctuating axial and proximal limb rigidity, lumbar hyperlordosis, postural instability, gait difficulties, falls, painful muscular cramps, spasms, and hyperactivity to stimuli [10,11]. Depression and generalized anxiety disorder are also common, sometimes even predominate [12]. Apart from classic and paraneoplastic forms of SPS, there are numerous variant forms: focal, segmental; progressive encephalomyelitis with rigidity and myoclonus; jerky SPS; and SPS comorbid with ataxia and epilepsy [10]. Diagnosis of SPS is based on the 2009 Dalakas criteria, electromyography examination (continuous motor unit activity at rest), and detection of specific antibodies [13].

Standard symptomatic medical management includes GABA-ergic agonists (diazepam, clonazepam), baclofen, anticonvulsants, and physical therapy [14]. First-line immunomodulatory therapy includes corticosteroids, intravenous immunoglobulins (IVIGs) [15], and therapeutic plasma exchange (TPE) [16]. Second-line immunomodulatory therapy includes cyclophosphamide, mycophenolate mofetil, and rituximab [17].

The case presents an anti-GAD65-positive patient with marked deterioration of symptoms, in whom TPE was used successfully as an adjunct therapy. We performed a review of the available literature in the context of safety and efficacy of TPE in acute exacerbation of SPS.

## 2 Case presentation

A 49-year-old female patient with exacerbation of SPS was admitted to the intensive care unit (ICU) to undergo TPE. The patient was diagnosed with SPS in 2013, the first symptoms noticed were pain in the lower extremities with paroxysmal increase in muscle tone; forced, painful, upright positioning of the left limbs; and two falls due to sudden generalized stiffness triggered by external stimuli. The comorbidities included type 2 diabetes mellitus, Hashimoto’s thyroiditis, and dyslipidemia. Past medical history, along with adverse effects that the patient developed, is presented in Table 1.

**Table 1:** Adverse effects to standard medical therapy and maximal tolerated dosing

Medication	Adverse effect	Max. daily tolerated dose (mg)
Diazepam	Vertigo, tachycardia, hypotension	14
Clonazepam	Worsened dystonia, speech difficulties	None
Baclofen	Increased rigidity	None
Gabapentin	Vertigo, tachycardia, hypotension	1,800
Levetiracetam	Paradoxical convulsions	1,500
Methylprednisolone	Hyperglycemia	Not tolerating therapeutic doses (1,500)

In 2018 and 2019, the patient had the erector spine muscle injected with botulinum toxin A (1,000 U), what reduced spasticity and improved patient’s gait. Over the previous 6 months, despite fixed daily oral doses of diazepam and gabapentin, the patient reported progression of symptoms: nystagmus, paresis, and spasticity of the left extremities; pain in the left lower extremity; paroxysmal axial rigidity; falls; dysphagia; and dyspnea. Due to exacerbation of the disease, the patient was admitted to the department of neurology for medical stabilization. IVIG was not used as it is not reimbursed by the Polish national healthcare insurer for the treatment of immune-mediated conditions. After an unsuccessful attempt at medical stabilization (5 days), the patient was admitted to the ICU to undergo TPE.

At the time of admission to the ICU, the concentration of anti-GAD65 was >2,000 U mL^−1^. Following admission to the ICU, the left radial artery was cannulated (Arterial Cannula 20 G; Becton Dickinson, Poland) and continuous, invasive arterial blood pressure monitoring was started along with minimally invasive pulse contour hemodynamic monitoring (LiDCO Rapid; LiDCO, United Kingdom). The right internal jugular vein was cannulated under ultrasound guidance (M7; Mindray, People’s Republic of China) and a 13 Fr dialysis catheter (GAMCATH HighFlow Dolphin Catheter; Baxter, Poland) was inserted. A standard continuous renal replacement therapy multifiltrate system (Fresenius Medical Care, Germany) along with plasmaFlux P2dry plasma filter (Fresenius Medical Care, Germany) was used. We used unfractionated heparin for anticoagulation and 4% human albumin as substitution fluid: a mixture of 20% human albumin and multiBic solution (Fresenius Medical Care) in the 1:5 ratio. The plasma volume exchanged was 1.5. We planned and performed 5 every-other-day procedures, no complications were reported. In the ICU, further two episodes of painful (pain sensation described as worse than labor pains) stiffness with muscle contractions occurred: at the time of a dialysis catheter insertion and spontaneously on day 2 of the ICU stay. Following the fourth procedure, a noticeable reduction in paresis/dystonic posturing and improved gait were noticed. The findings from neurological examination before and immediately following the course of TPE are presented in Table 2.

**Table 2:** Neurologic symptoms before and following the course of therapeutic plasma exchange

Neurological symptoms	Before therapeutic plasma exchange	After therapeutic plasma exchange
Nystagmus	Transient, low-phase, horizontal	Absent
Spasticity	Left limbs, paraspinal (hyperlordosis)	Significant improvement
Dystonia (left foot), UDRS	3	1
Dystonia (left foot), GDS	8	4
Gait, TUG	52 s (walking frame)	23 s (no support)
Muscle strength, mBMRC		
Left upper limb	4	4/5
Left lower limb	2/3	4
Right lower limb	4/5	5

mBMRC – modified British Medical Research Council Scale, GDS – global dystonia severity rating scale, TUG – timed up and go, UDRS – unified dystonia rating scale.

The total hospital length of stay was 14 days. The concentration of anti-GAD65 stayed elevated following TPE (>2,000 U mL^−1^ twice after the completion of the last TPE), nevertheless improvement in clinical symptoms remained (12-month follow-up period).

Due to the noninterventional design of the study, the Bioethics Committee approval was not needed. All patients’ data were obtained in accordance with the national law regulations of personal data management, after the written informed consent was given by the patient on hospital admission. The project was approved by the head of the institution and departments within which the work was undertaken; it conforms to the provisions of the Declaration of Helsinki (as revised in Fortaleza, Brazil, October 2013).

## 3 Discussion

We present a case report of a patient diagnosed with a typical form of SPS with a high titer of anti-GAD65 (>2,000 U mL^−1^) and typical comorbidities (diabetes mellitus and thyroiditis). The patient experienced an exacerbation of SPS with an unsuccessful attempt at pharmacological stabilization. As an adjunct therapy, we performed TPE (ICU setting), which led to significant clinical improvement (Table 2). No complications of the procedure itself reported.

The role of TPE in SPS has not been clearly defined. The most recent guidelines from the American Society for Apheresis report on 367 SPS patients (6 case series: 344 patients; 21 case reports: 23 patients) [18]. The rationale for the use of TPE in SPS is based on mixed results of TPE treatment provided by individual physicians who came across the syndrome. In most cases, TPE was used as an adjunct to immunosuppressive therapy. Relief of the SPS symptoms with TPE, even partial, was reported in 50–60% of patients [18]. That is the reason why the American Society for Apheresis in its recent guidelines did not make a strong recommendation for TPE in SPS (Grade 2C, category III) [18]. Since then Albahra et al. showed that in 10 patients with acute exacerbation of SPS (8 of whom were anti-GAD65 positive), TPE led to complete resolution of disease in 3 patients, partial resolution in 5 patients, and 2 patients showed no improvement [19]. Our case report is in line with the previous reports showing that TPE might be beneficial in anti-GAD65-positive SPS patients. The summary of the published case series and case reports, in the context of TPE efficacy in SPS, is presented in Table 3.

**Table 3:** Efficacy and safety profile of therapeutic plasma exchange in exacerbation of SPS

Paper (author, year)	Anti-GAD65 positive/total cases	TPE frequency (procedure/time period)	TPE efficacy (number of patients)	Complications (number of patients)
Vicari et al., 1989 [22]	1/1	5/10 days	Improvement (1)	Not observed
Harding et al., 1989 [23]	2/2	5/5 days	No improvement (2)	Not observed
Gordon et al., 1991 [24]	1/1	Not reported	No improvement (1)	Not reported
Brashear and Phillips, 1991 [25]	1/1	Not reported	Improvement (1)	Not observed
Nakamagoe et al., 1995 [26]	0/1	4/8 days	Improvement (1)	Not observed
Fogan et al., 1996 [27]	0/1	6/14 days	Improvement (1)	Not observed
Barker et al., 1998 [28]	2/2	Not reported	No improvement (2)	Not observed
Hao et al., 1999 [29]	1/1	Chronic: 3/week, 2/week, 1/week	Improvement (1)	CLABSI (1)
Hayashi et al., 1999 [30]	0/1	4/8 days	Improvement (1)	Not observed
Shariatmadar and Noto, 2001 [31]	0/2	6/12 days	Improvement (1)	Not observed
		5/10 days	No improvement (1)	
De la Casa-Fages et al., 2012 [20]	2/2	1/week	Improvement (2)	Not observed
McKeon et al., 2012 [32]	79/99	Not reported	Improvement (1)	Not reported
Pagano et al., 2014 [33]	6/9	5/10 days (7 patients)	Improvement (7)	CLABSI (1)
		10/20 days (1 patient)	No improvement (2)	Hypotension (1)
		15/30 days (1 patient)		
Georgieva and Parton, 2014 [34]	1/1	5/5 days (1 patient)	Improvement (1)	Not observed
Zdziarski et al., 2015 [35]	1/1	2 courses	Improvement (1)	Low albumin (1)
				Anemia (1)
				Tachycardia (1)
Buechner et al., 2015 [10]	2/3	Attempt (1 patient)	No improvement (1)	Thrombosis (1)
Pham and Williams, 2016 [36]	2/2	30/60 days	Improvement (1)	Hypotension (1)
			No improvement (1)	
Albahra et al., 2019 [19]	8/10	5/10 days (10 patients)	Improvement (8)	Not observed
		Chronic (6 patients)	No improvement (2)	

Anti-GAD65 – antibodies to the 65-kD isoform of glutamic acid decarboxylase, CLABSI – central line-associated bloodstream infection, SPS – stiff person syndrome, TPE – therapeutic plasma exchange.

Based on the published literature (Table 3), out of the total number of 163 patients reported, 120 tested positive for anti-GAD65. Only 40 (25% of the total number) patients underwent TPE (30 seropositive and 10 seronegative for anti-GAD65). Following TPE, 28 (70%, 23 anti-GAD65-positive) patients showed marked improvement and 12 (30%, 7 anti-GAD65-positive) patients showed no improvement. Despite the concentration of anti-GAD65 following TPE may not decrease, clinical improvement is still possible [20].

In our patient, the concentration of anti-GAD65 also did not decrease following TPE (>2,000 U mL^−1^ a month after the completion of the last TPE), nevertheless improvement in clinical symptoms remained (12-month follow-up). Some authors suggest that the lack of effectiveness of TPE in SPS is due to the presence of antibodies other than anti-GAD65, such as anti-amphiphysin and anti-GABA_A_RAP. Anti-GAB_A_RAP is a 14-kD postsynaptic protein that inhibits GABA_A_ receptor expression in about 65% of SPS patients, making patients with anti-GABA_A_RAP respond better to IVIG compared to other treatments [21].

The safety profile of TPE in SPS has not been accurately explored. The possible TPE complications include those associated with dialysis catheter insertion (local hematoma, pneumothorax, and hemothorax) and maintenance (local infection, central line-associated bloodstream infection [CLABSI], deep vein thrombosis) and complications associated with the procedure itself (tachycardia, hypotension, dyspnea, hypoalbuminemia, citrate toxicity, allergic reaction, and decreased humoral immunity).

Based on the published literature (Table 3), the most frequent TPE complications were CLABSI (2 of 40 patients, 5%), hypotension (2 of 40 patients, 5%), citrate toxicity (1 of 40 patients, 2.5%), endocarditis (1 of 40 patients, 2.5%), thrombosis (1 of 40 patients, 2.5%), hypoalbuminemia (1 of 40 patients, 2.5%), and tachycardia (1 of 40 patients, 2.5%). In our opinion, infection is probably the most serious complication. It is a complication of the procedure itself during which patient’s serum (immunoglobulins) is replaced with an immunoglobulin-free solution. Moreover, the presence of a dialysis catheter is a risk factor for CLABSI. In order to reduce the risk infection, one should always exclude infection (in our case the patient’s C-reactive protein concentration before the procedure was 2 mg L^−1^). All other complications can be eliminated/minimized by a meticulous technique of dialysis catheter insertion (optimized coagulation profile, US guidance, aseptic technique, full barrier precautions, and skilled staff), proper maintenance of a dialysis catheter, and multimodal monitoring of a patient during procedure (ICU setting).

## 4 Conclusion

Therapeutic plasma exchange may be a useful adjunct therapy for exacerbation of SPS not responding to standard medical therapy. The frequency of complications seems low. The exact efficacy and safety profile of TPE in this clinical setting remains undetermined and requires further research.
